# Single Site Suppressors of a Fission Yeast Temperature-Sensitive Mutant in *cdc48* Identified by Whole Genome Sequencing

**DOI:** 10.1371/journal.pone.0117779

**Published:** 2015-02-06

**Authors:** Irina N. Marinova, Jacob Engelbrecht, Adrian Ewald, Lasse L. Langholm, Christian Holmberg, Birthe B. Kragelund, Colin Gordon, Olaf Nielsen, Rasmus Hartmann-Petersen

**Affiliations:** 1 Department of Biology, University of Copenhagen, Copenhagen, Denmark; 2 MRC Human Genetics Unit, Western General Hospital, Edinburgh, Scotland, United Kingdom; University of Cambridge, UNITED KINGDOM

## Abstract

The protein called p97 in mammals and Cdc48 in budding and fission yeast is a homo-hexameric, ring-shaped, ubiquitin-dependent ATPase complex involved in a range of cellular functions, including protein degradation, vesicle fusion, DNA repair, and cell division. The *cdc48^+^* gene is essential for viability in fission yeast, and point mutations in the human orthologue have been linked to disease. To analyze the function of p97/Cdc48 further, we performed a screen for cold-sensitive suppressors of the temperature-sensitive *cdc48-353* fission yeast strain. In total, 29 independent pseudo revertants that had lost the temperature-sensitive growth defect of the *cdc48-353* strain were isolated. Of these, 28 had instead acquired a cold-sensitive phenotype. Since the suppressors were all spontaneous mutants, and not the result of mutagenesis induced by chemicals or UV irradiation, we reasoned that the genome sequences of the 29 independent *cdc48-353* suppressors were most likely identical with the exception of the acquired suppressor mutations. This prompted us to test if a whole genome sequencing approach would allow us to map the mutations. Indeed genome sequencing unambiguously revealed that the cold-sensitive suppressors were all second site intragenic *cdc48* mutants. Projecting these onto the Cdc48 structure revealed that while the original temperature-sensitive G338D mutation is positioned near the central pore in the hexameric ring, the suppressor mutations locate to subunit-subunit and inter-domain boundaries. This suggests that Cdc48-353 is structurally compromized at the restrictive temperature, but re-established in the suppressor mutants. The last suppressor was an extragenic frame shift mutation in the *ufd1* gene, which encodes a known Cdc48 co-factor. In conclusion, we show, using a novel whole genome sequencing approach, that Cdc48-353 is structurally compromized at the restrictive temperature, but stabilized in the suppressors.

## Introduction

The Cdc48 protein is an abundant ATPase complex known in higher eukaryotes as p97 or valosin-containing protein (VCP) [1]. Structurally, the Cdc48 ATPase forms a ring-shaped, homohexameric, chaperone-like complex [2] [3] [4] [5] [6]. The monomer is a phylogenetically highly conserved protein. It contains an N-domain, used for co-factor binding [7], and two “ATPase associated with various activities” (AAA) modules, called D1 and D2, that couple coordinated ATP-hydrolysis to conformational changes of the hexameric complex [8]. The ATP-driven conformational changes allow Cdc48 to physically disassemble protein complexes and segregate proteins from their binding partners [9] [10]. This activity is essential for a number of cellular pathways, including membrane fusion [11], protein degradation [12] [13], DNA repair [14], and transcription factor maturation [15] [16]. The p97/Cdc48 protein is essential for viability, but single site variants in human p97 have been linked to amyotrophic lateral sclerosis (ALS) [17] and to the multiple-disorder known as inclusion body myopathy associated with Paget's disease of the bone and frontotemporal dementia (IBMPFD) [18] [19].

Although Cdc48 is able to bind ubiquitin and ubiquitylated proteins directly [20], most Cdc48 functions require various Cdc48 cofactors [21]. Functionally, these cofactors are diverse and each probably directs Cdc48 to a particular cellular function. For instance, the cofactors p47 and Ubxd7 direct p97 to functions in membrane fusion [11] and protein degradation [22], respectively, while the Ufd1 cofactor appears to be more broadly involved in Cdc48 function. Thus, the Ufd1/Npl4 heterodimer is well known to function in the degradation of misfolded proteins derived from endoplasmic reticulum (ER) via the so-called ER-associated degradation (ERAD) pathway [4], but more recently Ufd1 has also been linked to the DNA damage response [23] [24] [25], to negative regulation of the Aurora B kinase [26], and to the degradation of aberrant nascent polypeptides bound to the ribosome [27]. To support these functions, Ufd1 appears to recruit ubiquitylated and/or sumoylated substrates to Cdc48-powered extraction from other proteins, lipids, or chromatin.

Here we describe a screen for cold-sensitive (cs) suppressors of the *cdc48–353* temperature-sensitive (ts) mutant in fission yeast [28] [29] carrying a single G338D point mutation in the conserved *cdc48* gene. In total, we isolated 29 spontaneous suppressors of the *cdc48–353* temperature-sensitive phenotype, of which 28 displayed a cs growth defect, while one did not. Whole genome sequencing surprisingly revealed that all the cs pseudo revertants were intragenic second site suppressors. Mapping the suppressor mutations on the Cdc48 structure revealed that while the original G338D mutation is located near the central pore in the Cdc48 hexameric ring, most of the suppressor mutations are located at the subunit-subunit and inter-domain boundaries. This suggests that the Cdc48–353 hexamer is structurally or dynamically compromised at the restrictive temperature, but re-established in the suppressor mutants. Whole genome sequencing of the extragenic *cdc48–353* suppressor revealed a frame shift mutation leading to an early stop codon in the *ufd1* gene, encoding the Cdc48 co-factor Ufd1.

## Materials and Methods

### *S*. *pombe* strains and techniques

Fission yeast strains used in this study (Table 1) are derivatives of the wild type heterothallic strains *972h* ^*-*^ and *975h* ^*+*^. Some strains were purchased from the fission yeast knock-out collection (Bioneer). Standard genetic methods and media were used and *S*. *pombe* transformations were performed using lithium acetate [30].

**Table 1:** Fission yeast strains used in this study.

Strain	Genotype	Reference
wild type	leu1–32 ura4-D18	Lab stock
cdc48–353	cdc48–353 leu1–32 ura4-D18	[29]
cdc48–353-flag	cdc48–353-flag leu1–32 ura4-D18	[29]
*cdc48–353* ^sup^ *1–29*	*cdc48–353* ^sup^ *1–29 cdc48–353 leu1–32 ura4-D18*	This study
*SPBC16A3*.*10*Δ	SPBC16A3.10::G418 leu1–32 ura4-D18	Bioneer
*SPBC16A3*.*06*Δ	SPBC16A3.06::G418 leu1–32 ura4-D18	Bioneer

### Screening the *cdc48–353* allele for pseudo revertants

Screening was performed essentially as described previously in budding yeast [31]. Briefly, the *cdc48–353* (*cdc48-G338D*) strain [29] was grown at the permissive temperature (30°C) until mid-exponential phase, and then diluted 200-fold and distributed with 500 μL in each of 2000 tubes. These cultures were then incubated at the restrictive temperature (36°C) for 48 hours. From those cultures where visible growth had occurred, 100 μL was spread on YES agar plates that were incubated at the restrictive temperature until colonies appeared. Only one single colony was selected from each plate and further tested for growth at 36°C and 20°C. Clones that were clearly no longer temperature-sensitive, but instead had acquired a cold-sensitive phenotype were selected for further analyses.

### Growth assays

For the growth assays on solid media, the *S*. *pombe* strains to be assayed were grown to mid-exponential phase before 10^6^ cells were spotted onto solid media YES plates and incubated at the indicated temperature until colonies formed.

### Purification of genomic DNA for sequencing

The genomic DNA of the selected strains was purified by phenol/chloroform extraction. Briefly, cells from 5 mL overnight cultures were harvested by centrifugation (3000 g, 5 minutes) and resuspended into 1 mL buffer (1.2 M sorbitol, 50 mM sodium citrate, 50 mM sodium phosphate, 40 mM EDTA, pH 5.6) containing 2 mg/mL glucanex (Novozymes). After incubation for 45 minutes at 36°C, spheroblasts were isolated by centrifugation (3000 g, 5 minutes) and resuspended in 450 μL TE buffer (10 mM Tris/HCl, 1 mM EDTA, pH 8.0). The spheroblasts were lysed by adding 50 μL of 10% (w/v) SDS and incubating for 5 minutes at RT. Then 150 μL of 5 M potassium acetate was added and the mixture incubated on ice for 5 minutes, before centrifugation at 20000 g for 10 minutes. The entire supernatant was transferred to a fresh tube before addition of 650 μL of isopropanol. After centrifugation at 15000 g for 5 minutes, the precipitate was washed three times with 70% (v/v) ethanol and dried in a vacuum centrifuge at 30°C. The precipitate was then resuspended in 150 μL TE buffer containing 0.1 mg/mL RNase A (Sigma) and incubated at 37°C for 20 minutes. Then the preparations were extracted once with phenol:chloroform:isoamylalchohol 25:24:1 (BDH) and once with chloroform (Sigma). Finally, the DNA was precipitated by adding 25 μL 3 M sodium acetate pH 5.2 and 675 μL 96% ethanol and resuspended into 100 μL of TE buffer.

### Whole genome sequencing

The purified DNA from the four selected suppressor strains was sequenced as 100 bp (94 bp genomic fragment + 6 bp barcode) single-end using the Illumina HiSeq2000 sequencing technology. The samples were multiplexed in one lane according to the standard Illumina protocol. A total of 131.4 million reads was obtained, giving between 13 and 36 million reads for each sample, corresponding to an average coverage between 102 and 276 fold/bp.

### Quality control, mapping and post-processing

The reads were filtered such that the phred quality score was at least 30 in 80% of the bases. We used fastqc and FASTX-toolkit for quality assessment and filtering. The sequences were aligned to the *S*. *pombe* reference genome [32] using Bowtie2 [33], and post-processed with SAMtools [34].

### Variant calling and filtering procedure

Variant calling was carried out using the Bayesian statistics based GenomeAnalysisToolKit (GATK) [35], using the haploid option. We analyzed the original *cdc48–353* ts strain, and used the variants found as priors for running GATK on the suppressor strains. For comparison we also applied the simple threshold based variant detection algorithm VarScan [36] in combination with the SAMtools mpileup algorithm for variant calling.

### Structure analysis

The three-dimensional crystal structure of the full-length mouse p97 (PDB: 3CF3) [37] was used as a reference for the analysis of the mutated position and the sequences of mouse p97 and *S*. *pombe* Cdc48 were aligned by ClustalW2. Protonation states of side chains were calculated using PropKa (http://propka.ki.ku.dk) [38]. Structures were visualized by the PyMol Molecular Graphics System.

## Results

### Screening for spontaneous cold-sensitive suppressors of the *cdc48–353* mutant

**Fig 1:**
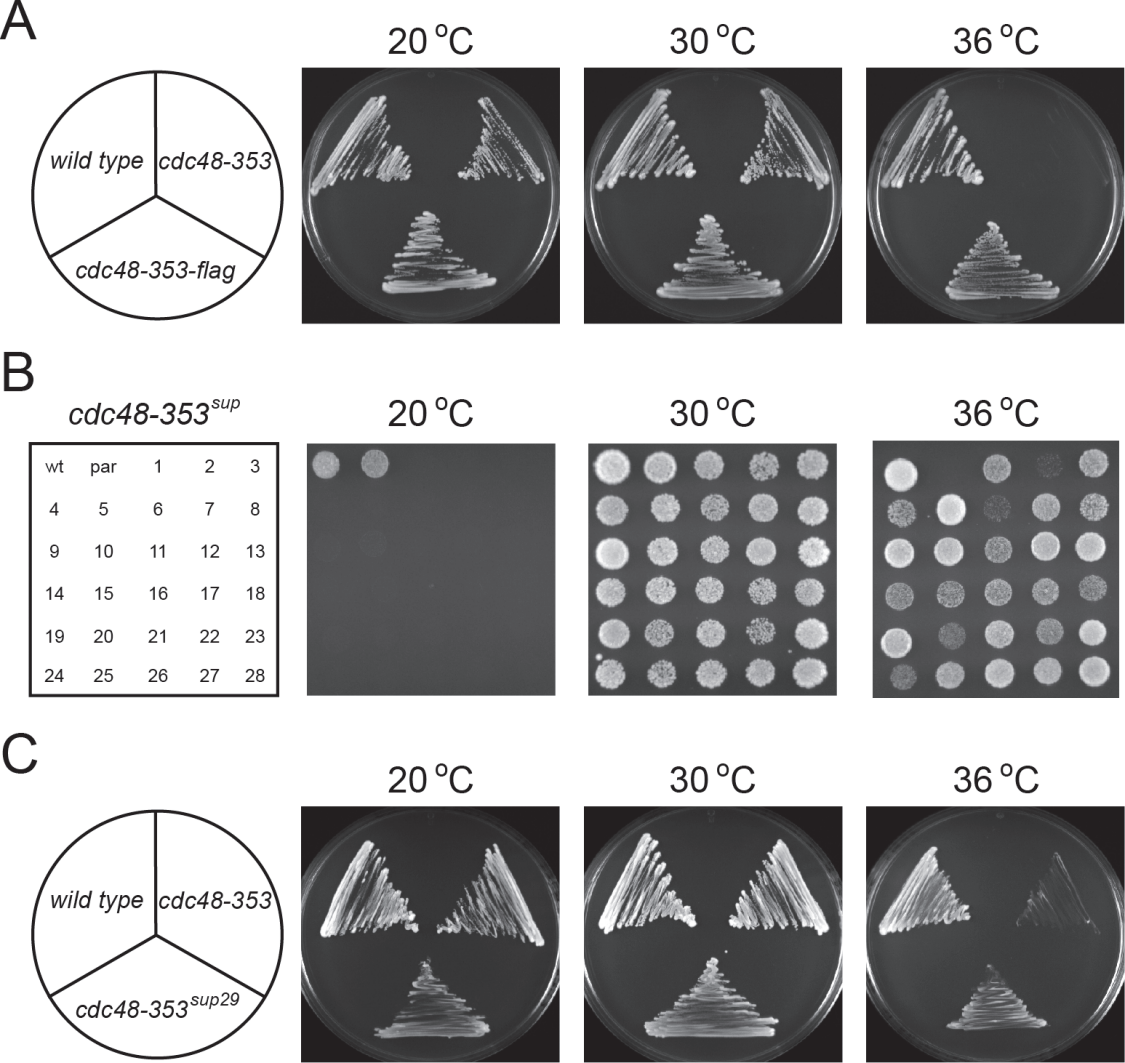
Growth phenotypes of the *cdc48–353* and *cdc48–353* ^sup^ strains. (A) Cell growth of wild type, *cdc48–353* and *cdc48–353-flag* strains on YES media was compared at the indicated temperatures. Note that the *cdc48–353* strain does not form colonies at 36°C. (B) Growth of wild type (wt), the *cdc48–353* parental strain (par) and the 28 different *cdc48–353* ^sup^ strains on YES media was compared at the indicated temperatures. For each strain 10^6^ cells were applied to the media. (C) Cell growth of wild type, *cdc48–353* and the *cdc48–353* ^*sup*^ *-29* strain on YES media was compared at the indicated temperatures.

To isolate suppressors of the *cdc48–353* strain, we performed a pseudo reversion screen with the *cdc48–353* temperature sensitive (ts) strain. For this strain, growth appeared normal at low temperature (20°C) and at the permissive temperature of 30°C, but was severely compromised at the restrictive temperature of 36°C (Fig. 1A). However, introduction of a flag epitope in the 3’ end of the *cdc48–353* reading frame caused loss of the ts phenotype (Fig. 1A). This observation suggests that even slight perturbations in the Cdc48 structure and/or co-factor binding may suppress the ts phenotype, making the *cdc48–353* strain ideally suited for a sensitive screen for suppressors of the ts phenotype. The *cdc48–353* mutant was therefore inoculated in 2000 separate tubes and incubated at the restrictive temperature (36°C). After 48 hours, growth was apparent in approximately 500 of the tubes, suggesting that independent spontaneous suppressor mutations had occurred in about 25% of the cultures. To ensure that no identical clones, but only independent suppressor mutants, were isolated, cells from each culture were spread on solid media plates and only one single colony from each plate was selected for further analyses. Since this screen had yielded such a high return, i.e. approximately 500 individual suppressor mutants, we decided to assess these suppressors for gain of a cold-sensitive phenotype, thereby limiting the number of strains while increasing the probability of isolating suppressors in essential genes. Out of the approximately 500 suppressor mutants, we found 29 individual strains that had lost the ts growth phenotype, but gained a cold-sensitive (cs) phenotype. When retesting for the cs phenotype, we found that 28 of the suppressors were truly cold-sensitive (Fig. 1B), and we named these strains *cdc48–353* ^sup^ *-1–28*. Upon retesting, one *cdc48–353* suppressor (*cdc48–353* ^sup^ *-29*) turned out not to be cs (Fig. 1C). This was also included in the subsequent analyses. Not all suppressors suppressed the ts phenotype equally well (Fig. 1B). Hence, *cdc48–353* ^sup^ *-2*, *6*, *18*, *20* and *24* only weakly suppressed the ts phenotype, while the others more strongly suppressed the ts phenotype (Fig. 1B).

### Whole genome sequencing

Mapping the mutations, leading to the ts suppressing and cs phenotype in the isolated clones, would traditionally require transformation with a genomic or cDNA library and isolation of clones, losing the cs phenotype (surviving at 20°C). Since the isolated *cdc48–353* suppressors were all spontaneous mutants, and not the result of mutagenesis induced by chemicals or UV irradiation, we reasoned that the genomic sequence of the 29 independent *cdc48–353* suppressors was most likely identical with the exception of the ts suppressing mutations. This reasoning, coupled with the recent advances in high-throughput DNA sequencing technologies making them feasible both in cost, precision and speed as standard laboratory procedures, inspired us to test if a whole genome sequencing approach would allow us to identify single site variants. We therefore selected 5 of the 29 isolated suppressors for whole genome sequencing using Illumina HiSeq2000 high-throughput sequencing technology. All of these whole genome sequencing reactions were bar-coded and multiplexed in one lane. The sequence coverage ranged between 102 and 276 reads/bp. The sequencing datasets have been deposited at the European Nucleotide Archive (http://www.ebi.ac.uk/ena/data/view/PRJEB7979; accession numbers: ERS627715-ERS627720, study accession number: PRJEB7979). When comparing our datasets with the published *S*. *pombe* genome sequence, we noticed that our sequencing data appeared more similar to that reported by the Broad Institute [39], rather than that of the Sanger Centre [32]. However, as the main focus here was to identify the ts suppressing mutations, and the isolated suppressors were independent suppressors of the same parental strain, we decided to compare our genome sequencing dataset with each other rather than with the published reference genomes. In this manner irrelevant differences between our sequencing data and the published datasets would not veil the identification of the ts suppressing mutations. Comparisons of sequences obtained from the individual strains revealed a match between the genomes, except for the changes listed in Table 2.

**Table 2:** Whole genome sequencing of selected ***cdc48–353*** ^**sup**^ mutants.

Strain	Affected gene	Amino acid substitution	Codon change
*cdc48–353* ^sup^ *-16*	cdc48	P481R	CCT→CGT
*cdc48–353* ^sup^ *-17*	cdc48	P481R	CCT→CGT
*cdc48–353* ^sup^ *-18*	cdc48	Q781stop	CAA→TAA
*cdc48–353* ^sup^ *-19*	cdc48	P292L	CCT→CTT
*cdc48–353* ^sup^ *-29*	ufd1	I210HRLstop	ATC→CATC

### The *cdc48–353* ^*sup*^ *-29* strain is a frame shift mutant of *ufd1*

**Fig 2:**
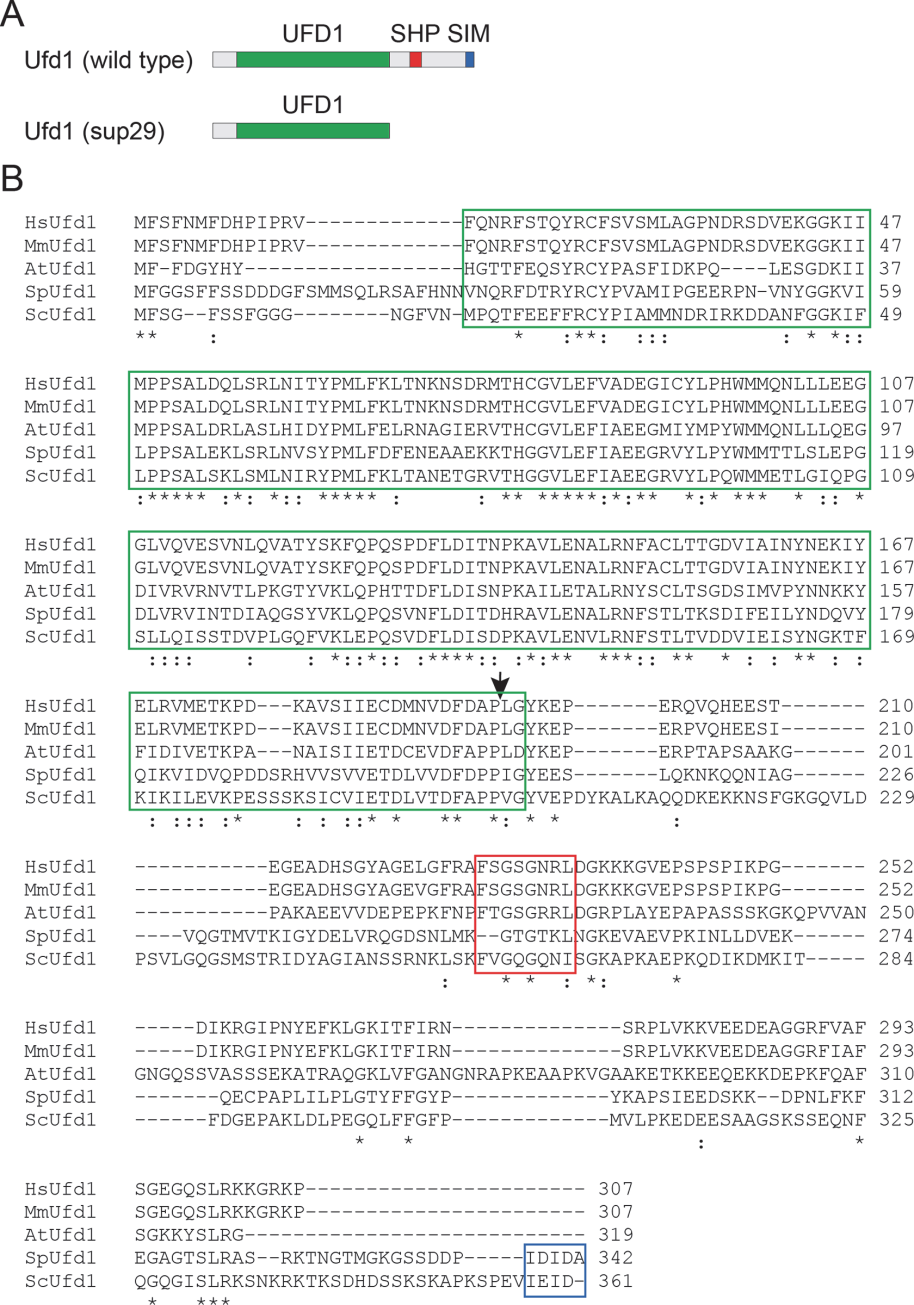
The *cdc48–353* ^sup^ *-29* suppressor mutation maps to *ufd1*. (A) Schematic representation of the Ufd1 domain organization. The Ufd1-domain is shown in green, the SHP box and SIM in red and blue, respectively. The effect of the frame shift mutation and early stop codon in suppressor 29 (sup29) is shown in the lower panel. (B) Multiple sequence alignment of the Ufd1 protein from human (Hs), mouse (Mm), plant (At), fission yeast (Sp) and budding yeast (Sc). The UFD1 domain is boxed in green, the SHP box in red and the SIM in blue. The position of the frame shift mutation is marked with an arrow. Identical and homologues residues have been marked with (*) and (:), respectively.

The whole genome sequencing of the *cdc48–353* ^sup^ *-29* suppressor suggested that the suppressing mutation had occurred in the *ufd1* gene (Table 2). To verify this result, we first PCR sequenced the *ufd1* and *cdc48* genes in this strain. This confirmed the results from the whole genome sequencing that the *cdc48–353* gene was unchanged while a cytosine was inserted in front of an ATC codon for isoleucine 210 in *ufd1*, leading to a frame shift terminating after another three residues (Ufd1 I210HRLstop) (Fig. 2A). To provide genetic evidence that this insertion was responsible for the observed suppression, the *cdc48–353* ^sup^ *-29* strain was crossed to mutants where the *SPBC16A3*.*10* (*MBOAT/ALE1*) and *SPBC16A3*.*06* (*TAD1*) genes were replaced with a G418 resistance cassette. Since *SPBC16A3*.*10* and *SPBC16A3*.*06* flank the *ufd1* ^+^ gene (-249 bp and +5018 bp away, respectively), strong linkage was expected. Indeed, we did not find any progeny carrying the *cdc48–353* ^sup^ *-29* mutation that were also resistant to G418, suggesting that the ts suppressing mutation was tightly linked to the *ufd1* locus. Together, this proves that the frame shift mutation in *ufd1* is responsible for suppressing the *cdc48–353* ts phenotype. Although the *ufd1* gene is essential for viability [40] [41], the frame shift mutation in *cdc48–353* ^sup^ *-29* did not display any obvious growth defect, despite that the truncated Ufd1-I210HRLstop protein entirely lacks both the SHP box and the SIM motif (Fig. 2B) involved in p97/Cdc48 and SUMO binding [23] [24] [25], respectively.

### All isolated pseudo revertants with the cs phenotype were intragenic second site suppressors

We were surprised that all of the four cs strains, analyzed by genome-wide sequencing, were intragenic, second-site suppressors of the original *cdc48–353* ts mutant (Table 2). This suggested that perhaps all 28 cold-sensitive ts suppressors were intragenic *cdc48* suppressors. To test this, we first crossed all the *cdc48–353* ^sup^ strains to wild type cells and analyzed the progeny for the occurrence of the ts phenotype. In none of the mutants did the ts and cs phenotypes segregate, indicating that the cs suppressor mutations had indeed all occurred near the *cdc48–353* locus. To determine if they were all intragenic second-site suppressors, the *cdc48* gene was PCR sequenced in all the cs *cdc48–353* ^sup^ strains. These PCR sequencing data confirmed the results from the whole genome sequencing, and revealed, as expected from the crossing experiments, that all the cs *cdc48–353* ^sup^ strains had acquired second-site intragenic *cdc48* mutations (Table 3). Some of these second site suppressor mutations were unique, while others were identified multiple times in independent suppressor strains (Table 3). For instance, the P481R mutation occurred in eight independent *cdc48–353* ^sup^ strains, the early stop codon at position Q781 occurred in three independent clones, while e.g. the V767A mutation was only observed in one clone (Table 3). For the P292, we found variants with either a threonine or more commonly a leucine residue at this position (Table 3). However, in all cases the suppressing mutations were caused by single nucleotide substitutions (Table 3). In addition, we noticed that the E598V and Q781stop mutants were less efficient in suppressing the ts phenotype than the other suppressor mutations (Fig. 1B and Table 3).

**Table 3:** All identified ***cdc48–353*** ^**sup**^ mutants.

Strain	Gene	Mutation	Codon	Sup. strength	Genome seq.
*cdc48–353* ^sup^ *-1*	cdc48	P481R	CCT→CGT	++	No
*cdc48–353* ^sup^ *-2*	cdc48	E598V	GAG→GTG	+	No
*cdc48–353* ^sup^ *-3*	cdc48	P481R	CCT→CGT	++	No
*cdc48–353* ^sup^ *-4*	cdc48	R369P	CGT→CCT	++	No
*cdc48–353* ^sup^ *-5*	cdc48	L524FL 715 Asn	TTG→TTT	+++	No
		K715N	AAA→AAT		
*cdc48–353* ^sup^ *-6*	cdc48	E598V	GAG→GTG	+	No
*cdc48–353* ^sup^ *-7*	cdc48	S359GP	AGT→GGT	++	No
		P481R	CCT→CGT		
*cdc48–353* ^sup^ *-8*	cdc48	R644H	CGT→CAT	++	No
*cdc48–353* ^sup^ *-9*	cdc48	N371S	AAC→AGC	+++	No
*cdc48–353* ^sup^ *-10*	cdc48	P516S	CCT→TCT	+++	No
*cdc48–353* ^sup^ *-11*	cdc48	R764S	CGC→AGC	++	No
*cdc48–353* ^sup^ *-12*	cdc48	P292L	CCT→CTT	+++	No
*cdc48–353* ^sup^ *-13*	cdc48	P655S	CCC→TCC	+++	No
*cdc48–353* ^sup^ *-14*	cdc48	R644H	CGT→CAT	++	No
*cdc48–353* ^sup^ *-15*	cdc48	P516L	CCT→CTT	++	No
*cdc48–353* ^sup^ *-16*	cdc48	P481R	CCT→CGT	++	Yes
*cdc48–353* ^sup^ *-17*	cdc48	P481R	CCT→CGT	++	Yes
*cdc48–353* ^sup^ *-18*	cdc48	Q781stop	CAA→TAA	+	Yes
*cdc48–353* ^sup^ *-19*	cdc48	P292L	CCT→CTT	+++	Yeshjjhj
*cdc48–353* ^sup^ *-20*	cdc48	Q781stop	CAA→TAA	+	No
*cdc48–353* ^sup^ *-21*	cdc48	V767A	GTC→GCC	+++	No
*cdc48–353* ^sup^ *-22*	cdc48	P481R	CCT→CGT	++	No
*cdc48–353* ^sup^ *-23*	cdc48	P655S	CCC→TCC	+++	No
*cdc48–353* ^sup^ *-24*	cdc48	Q781stop	CAA→TAA	+	No
*cdc48–353* ^sup^ *-25*	cdc48	P481R	CCT→CGT	+++	No
*cdc48–353* ^sup^ *-26*	cdc48	P292T	CCT→ACT	+++	No
*cdc48–353* ^sup^ *-27*	cdc48	P481R	CCT→CGT	+++	No
*cdc48–353* ^sup^ *-28*	cdc48	E325K	GAG→AAG	+++	No
*cdc48–353* ^sup^ *-29*	ufd1	I210HRLstop	ATC→CATC	++	Yes

Collectively, these data suggest that amino acid replacements at multiple positions in Cdc48 may remedy for the original Cdc48 G338D substitution, while if any extragenic cs suppressors of the *cdc48–353* phenotype exist, these are extremely rare.

### The *cdc48–353* suppressors may confer structural stability to the Cdc48 hexamer

**Fig 3:**
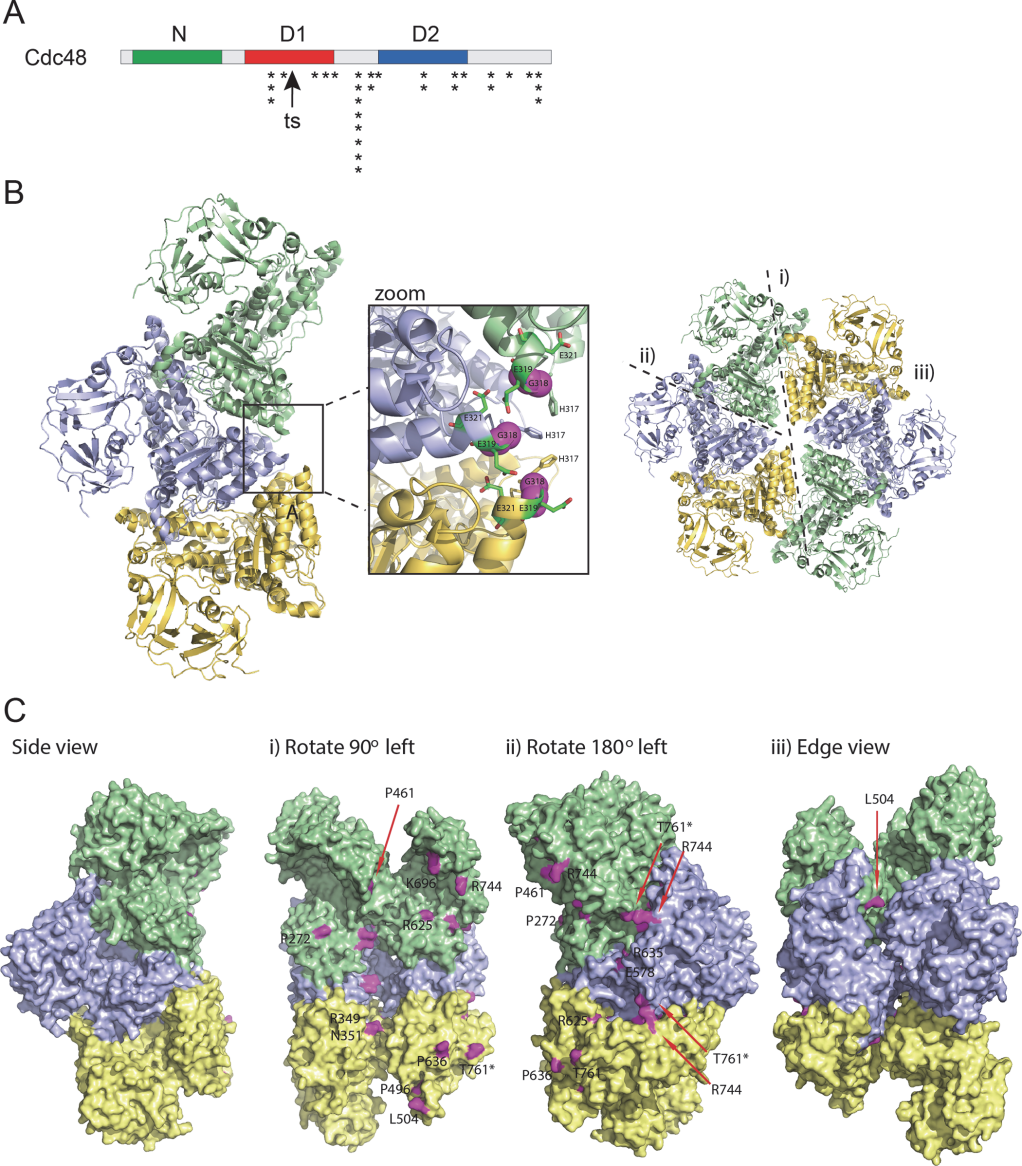
The *cdc48–353* ^sup^ *-29* suppressor mutation maps to *ufd1*. (A) Schematic representation of the Cdc48 domain organization. The N-domain is shown in green, and the D1 and D2 ATPase domains in red and blue, respectively. The site of the ts G338D mutation is marked with an arrow. The suppressor mutations are marked with an asterisk. (B) Overview of the hexameric organization of p97/Cdc48 indicating the individual subunits in green, yellow and blue, respectively with three different views shown by dashed lines (marked by i), ii) and iii)). Only one half of the hexameric ring is illustrated. The zoom window highlights the position of the SpG338D (MmG318D) mutation located in the D1 ATPase domains facing the central pore of the hexameric ring (shown by magenta spheres) in an area with MmE319 (SpE339) MmE321 (SpE341), and a histidine cluster formed by MmH317 (SpN337). These side chains are shown by sticks. (C) Positions of the inter-facial suppressor mutations at the three different views highlighted in (B). Numbering is according to the mouse sequence (MmP272∼SpP292; MmR349∼SpR369; MmN351∼SpN371; MmP461∼SpP481; MmP496∼SpP516; MmL505∼SpL524; MmE578∼SpE598; MmR625∼SpR644; MmP636∼SpP655; MmK696∼SpK715; MmR744∼SpR764; MmT761(stop) ∼SpQ781(stop)). MmP496∼SpP516 is completely buried and not visible.

In an attempt to understand the positioning of the intra-*cdc48* ts suppressing variants, we first mapped their positions on the domain organization of Cdc48 (Fig. 3A). Intriguingly, most of the suppressors clustered to the C-terminal part of Cdc48, including the linker region between the ATPase domains and in the D2 ATPase domain. None of the suppressor mutations localized to the N-domain.

The structure of the mouse Cdc48 orthologue, p97, has been solved to a resolution of 4.25 Å (PDB: 3CF3) [37], revealing a symmetric hexameric ring (Fig. 3B). Using this structure together with a sequence alignment of the *Mus musculus* (Mm) and *S*. *pombe* (Sp) sequences (S1 Fig.), we marked the position of the residues corresponding to the *S*. *pombe* original G338D (MmG318) ts lesion and the suppressors onto the three-dimensional structure. The SpG338D (MmG318D) mutation is located in the D1 ATPase domain facing the central pore of the hexameric ring in an area with a glutamate at position 319 (SpE339) and a histidine cluster formed by MmH317 (SpN337) as well as an additional acidic residue MmE321 (SpE341) (Fig. 3B). From calculation of pKa values of these residues by PropKa, using the coordinates of mouse p97 (PDB: 3CF3), MmH317 is uncharged at neutral pH (pKa 6.28) and MmE319 and MmE321 both negatively charged (pKa 4.64 and 4.95, respectively). Thus, these residues form a distinct acidic center. Introduction of an additional acidic residue in this confined space would therefore be anticipated to have two possible effects, depending on the protonation state of its side chains. Either, and most likely, the introduction of a charge would significantly destabilize the quaternary structure of the complex or, if the side chain is uncharged, this could instead lead to a stabilization of the oligomer. In that case, such an oligomeric conformation would be less able to respond to ATP-driven allosteric conformational changes. On these grounds, it is therefore highly interesting to note that most of the suppressor mutations located to subunit-subunit boundaries (Fig. 3C) and in loops between the domains (inter-domain surfaces) (Fig. 3C), while the outer surface of Cdc48 appeared completely unchanged (Fig. 3C). The nature of the changes typically led to an increase in flexibility by proline substitutions (SpP292L/MmP272L, SpP481R/MmP461R, SpP516S/MmP496S, SpP655S/MmP636S) and glycine insertions (SpS359G/MmA339G), but also charge reversals (SpE325K/MmE305K) and neutralization of charges (SpR369P/MmR349P, SpR764S/MmR744S, SpE598V/MmE578V, SpK715N/MmK696N) were observed. Since the resolution of the structure is limited to 4.25 Å, an analysis of the atomic details of the interactions would be too speculative, but the distinct location between subunits strongly suggest that the reverting mutations act to either increase the stability of the hexameric state or act to increase structural flexibility needed for functional allosteric changes in the hexamer.

In conclusion, these data suggest that the Cdc48–353 hexamer is structurally compromized at the restrictive temperature being either destabilized or restricted from allosteric coupling, and that the suppressors function by re-establishing functionality through formation of optimized contacts at the subunit-subunit and inter-domain interfaces.

## Discussion

Here, we describe a screen for pseudo revertants of the temperature-sensitive *cdc48–353 S*. *pombe* strain, similar to screens performed previously in budding yeast [31]. With the advance of robotics and libraries of knock-out mutants, such screens have largely been replaced with various high throughput approaches such as the epistasis miniarray profiling (E-MAP) technique [42]. The readout of such methods are normally either no effect, synthetic rescue, or synthetic sick, and the data generated are therefore readily interpretable and do not require laborious cloning steps to map the interacting genes [43]. However, a shortcoming of these methods is that mutants in essential genes are only rarely identified.

As proven by our identification of the suppressing mutations in the *cdc48–353* ^*sup*^ strains, the whole genome sequencing strategy described here provides a simple, cost effective and powerful approach to identify single site variants, thus bypassing the tedious cloning steps involved when mapping the mutated genes. More importantly, this sequencing approach also allows for identification of mutations that do not result in any dramatic phenotypes, such as the *cdc48–353* ^*sup*^ *-29*, which we mapped to the *ufd1* gene. When we designed the suppressor screen, we chose to focus on mutations that simultaneously had acquired a cs phenotype, partly because this would provide a means of subsequently identifying the mutated genes by complementation. However, in retrospect this decision appears to have constrained the outcome of the screen to intragenic suppressors. Hence, it is likely that analyses of non-cs suppressors in addition to *cdc48–353* ^*sup*^ *-29* will define more extragenic suppressors that may shed new light on Cdc48 function. The lesion in the *cdc48–353-*suppressing *ufd1* mutant results in an early stop codon, effectively deleting about 40% of the essential Ufd1 protein. Intriguingly, this truncated Ufd1 lacks both the Cdc48-interacting SHP box and the C-terminal SUMO interaction motif (SIM). Previous studies have shown that the SUMO binding activity of Ufd1 is not essential in fission yeast [24] [23], and as is evident from our alignment (Fig. 2B), the C-terminal SIM is also not conserved to Ufd1 orthologues in higher eukaryotes. However, since Ufd1 has so far only been described as a Cdc48 co-factor, it is surprising that deletion of the Ufd1 SHP box is of no consequence to cell viability. This suggests that either the essential function of Ufd1 does not require association with Cdc48 or that perhaps Ufd1 has other means to interact with Cdc48, e.g. via Npl4.

When mapping the *cdc48–353* ^*sup*^ *-29* to the *ufd1* gene, we were also surprised to learn that exactly the same mutation, the insertion of a cytosine in front of the ATC codon for isoleucine 210 in *S*. *pombe ufd1*, has previously been isolated in a screen for mutants resistant to the adenosine analogue cordycepin (3'-deoxyadenosine) that inhibits growth and causes aberrant cell morphology [40]. This suggests that the sequence at this particular position of the *S*. *pombe* genome is somehow prone to mutation. It is tempting to speculate that such a mutational hotspot could be of functional importance. During evolution, mutations at this position could lead to formation of various protein products all carrying the N-terminal UFD1 domain fused to different C-terminal modules. Indeed, database searches for UFD1 domains, revealed that several species harbor proteins with an N-terminal UFD1 domain fused to different C-terminal domains (data not shown). However, it is not possible to determine if this is a consequence of a mutational hotspot.

Our genetic data and structural analyses on the intragenic *cdc48–353* suppressors clearly revealed that while the original ts lesion in *cdc48–353* is positioned in the central pore of the hexameric complex, most of the ts suppressing second-site mutations that we isolated cluster to subunit-subunit interfaces. This strongly suggests that the original ts lesion confers some structural instability to the protein complex that can be alleviated by modulating and thereby enhancing the inter subunit contacts. Accordingly, all these suppressors also displayed a strong cs phenotype. Perhaps the cs *cdc48* suppressors under cold conditions possess a lowered flexibility of the Cdc48 hexamer, leading to an impaired or complete loss of chaperone function. Previous size exclusion chromatography studies on the Cdc48–353 protein have shown that even at the restrictive temperature the mutant protein is still hexameric [28], suggesting that the structural destabilization we propose here does not cause a complete complex disassembly, but more likely compromise functional dynamics and will thus require more sensitive biophysical or structural techniques to be analyzed further.

Three independent intragenic *cdc48–353* suppressors that we isolated contained a premature stop codon at amino acid position 781. The truncated version of the Cdc48 protein, resulting from this mutation, lacks the last 35 residues, including the PUB/PUL domain binding site (PBS) [21], which should generate a Cdc48 protein unable to interact with Lub1 (Doa1/Ufd3 in budding yeast). However, since *lub1*Δ cells are not cold-sensitive (our unpublished results), the cs phenotype of the Cdc48 truncation is more likely to be attributed to a reduced structural flexibility of the hexameric complex. Indeed, this C-terminal region of Cdc48 is predicted to be highly flexible, disordered, and does not resolve in the p97 crystal structure [37].

Considering that all the isolated intragenic *cdc48–353* suppressors probably function either by stabilizing the Cdc48–353 structure, or by rescuing its dynamic responsiveness, it is likely that the Ufd1 truncation in a similar manner positively affects Cdc48–353 stability. The ts lesion in the Cdc48–353 protein is located in the pore of the hexameric ring, an area which is covered by a structurally highly dynamic interaction with the Ufd1/Npl4 heterodimer [44]. Although the N-terminal UFD1 domain is tightly folded [45], the C-terminal region of Ufd1 is hydrophilic and predicted to be intrinsically disordered (data not shown). When losing this disordered region, the mutant Ufd1 protein could perhaps reestablish some structural stability to the Cdc48–353 complex.

As mentioned, single amino acid substitutions in human p97/Cdc48 have been linked to ALS and the multiple disorder known as IBMPFD. In general, these clinical p97/Cdc48 mutations cluster to the N-domain of p97/Cdc48, an area that was found to be unaffected here. However, recent studies suggest that the molecular mechanism by which the IBMPFD relevant mutations affect chaperone function is linked to an altered inter-subunit communication, suggesting that tight inter-subunit coordination is required for efficient p97 function [19]. The results presented here on fission yeast Cdc48 support this notion.

## Conclusions

In conclusion, we show, using a novel whole genome sequencing approach to identify suppressor mutants, that Cdc48–353 is structurally compromized at the restrictive temperature, but stabilized in the suppressors.

## Supporting Information

S1 Fig Sequence alignment of p97 and Cdc48. ClustalW sequence alignment of mouse p97 and fission yeast Cdc48. Identical (*) and similar (:/.) residues have been marked.
(PDF)
