# Supplementary material for: Single Site Suppressors of a Fission Yeast Temperature-Sensitive Mutant in cdc48 Identified by Whole Genome Sequencing
Source: PLoS One. 2015 Feb 6;10(2):e0117779. doi: 10.1371/journal.pone.0117779 (PMC4319823; doi:10.1371/journal.pone.0117779)
Supplement: S1 Fig — ClustalW sequence alignment of mouse p97 and fission yeast Cdc48. Identical (*) and similar (:/.) residues have been marked. (PDF) [file pone.0117779.s001.pdf]

```
M. musculus      ---MASGADSK-----------------GDDLSTAILKQKNRPNRLIVDEAINEDNSVVS 40
S. pombe         MNAPSTMTDKKPEVEHLQGENPPKDTYSAEDTATAILRKKRKPNSLVVDDATNDDNSVIT 60
                     :: :*.*                 .:* :****::*.:** *:**:* *:****::

M. musculus      LSQPKMDELQLFRGDTVLLKGKKRREAVCIVLSDDTCSDEKIRMNRVVRNNLRVRLGDVI 100
S. pombe         LSSNTMETLQLFRGDTVVVKGKRRKDTVLIVLTDEEMEDGVARINRVVRNNLRVRLGDIV 120
                 **. .*: *********::***:*:::* ***:*:  .*   *:**************::

M. musculus      SIQPCPDVKYGKRIHVLPIDDTVEGITGNLFEVYLKPYFLEAYRPIRKGDIFLVRGGMRA 160
S. pombe         TINPCPDIKYAERISVLPLADTVEGLTGSLFDVYLKPYFVEAYRPIRKGDLFVVRGSMRQ 180
                 :*:****:**.:** ***: *****:**.**:*******:**********:*:***.**

M. musculus      VEFKVVETDPSPYCIVAPDTVIHCEGEPIKREDEEESLNEVGYDDIGGCRKQLAQIKEMV 220
S. pombe         VEFKVVDVAPDEFGIVSQDTIIHWEGEPINREDEESSLAEVGYDDIGGCRRQMAQIRELV 240
                 ******:. *. : **: **:** *****:*****.** ***********:*:***:*:*

M. musculus      ELPLRHPALFKAIGVKPPRGILLYGPPGTGKTLIARAVANETGAFFFLINGPEIMSKLAG 280
S. pombe         ELPLRHPQLFKSIGIKPPRGILMYGPPGTGKTLMARAVANETGAFFFLINGPEIMSKMAG 300
                 ******* ***:**:*******:**********:***********************:**
                                                             *
M. musculus      ESESNLRKAFEEAEKNAPAIIFIDELDAIAPKREKTHGEVERRIVSQLLTLMDGLKQRAH 340
S. pombe         ESESNLRKAFEEAEKNSPAIIFIDEIDSIAPKREKTNGEVERRVVSQLLTLMDGMKARSN 360
                 ****************:********:*:********:******:**********:* *::

M. musculus      VIVMAATNRPNSIDPALRRFGRFDREVDIGIPDATGRLEILQIHTKNMKLADDVDLEQVA 400
S. pombe         VVVMAATNRPNSIDPALRRFGRFDREVDVGIPDPTGRLEILRIHTKNMKLADDVDLEQIA 420
                 *:**************************:****.*******:****************:*

M. musculus      NETHGHVGADLAALCSEAALQAIRKKMDLIDLEDETIDAEVMNSLAVTMDDFRWALSQSN 460
S. pombe         AETHGYVGSDLASLCSEAAMQQIREKMDMIDLDEDEIDAEVLDSLGVTMDNFRFALGSSN 480
                  ****:**:***:******:* **:***:***::: *****::**.****:**:**..**

M. musculus      PSALRETVVEVPQVTWEDIGGLEDVKRELQELVQYPVEHPDKFLKFGMTPSKGVLFYGPP 520
S. pombe         PSALRETVVEVPNVRWEDIGGLEEVKRELRETVQMPVMYAEKFLRFGVTPSKGVLFFGPP 540
                 ************:* ********:*****:* ** ** :.:***:**:********:***

M. musculus      GCGKTLLAKAIANECQANFISIKGPELLTMWFGESEANVREIFDKARQAAPCVLFFDELD 580
S. pombe         GTGKTLLAKAIANECSANFISVKGPELLSMWFGESESNVRDIFDKARAAAPCVVFLDELD 600
                 * *************.*****:******:*******:***:****** *****:*:****

M. musculus      SIAKARGGNIGDGGGAADRVINQILTEMDGMSTKKNVFIIGATNRPDIIDPAILRPGRLD 640
S. pombe         SIAKARGASAGDSGGG-DRVVNQLLTEMDGVNSKKNVFVIGATNRPDQIDPALMRPGRLD 659
                 *******.. **.**. ***:**:******:.:*****:******** ****::******

M. musculus      QLIYIPLPDEKSRVAILKANLRKSPVAKDVDLEFLAKMTNGFSGADLTEICQRACKLAIR 700
S. pombe         QLIYVPLPDEEARFSILQTQLRHTPVAEDVDLRAVAKATHGFSGADLEFVVQRAVKLAIK 719
                 ****:*****::*.:**:::**::***:****. :** *:*******  : *** ****:

M. musculus      ESIESEIRRERERQTNPS-AMEVEEDDPVPEIRRDHFEEAMRFARRSVSDNDIRKYEMFA 759
S. pombe         DSIEEDIKRENETGEAPADDVVMDEDASVSQVQRHHVEEAMKMARRSVSDAEVRRYEAYA 779
                 :***.:*:**.*    *:  : ::** .*.:::*.*.****::******* ::*:** :*

M. musculus      QT|LQQSRGFGSFRFPSGNQGGAGPSQGSGGGTGGSVYTEDNDDDLYG 806
S. pombe         HQ|LLTSRGLTGFQFDSADSNTNGPSFGN-----------DGADDLYA 815
                 : |*  ***: .*:* *.:..  *** *.           *. ****.
                   |STOP
```
